# Estimating the Impact of Newly Arrived Foreign-Born Persons on Tuberculosis in the United States

**DOI:** 10.1371/journal.pone.0032158

**Published:** 2012-02-27

**Authors:** Yecai Liu, John A. Painter, Drew L. Posey, Kevin P. Cain, Michelle S. Weinberg, Susan A. Maloney, Luis S. Ortega, Martin S. Cetron

**Affiliations:** 1 Division of Global Migration and Quarantine, Centers for Disease Control and Prevention, Atlanta, Georgia, United States of America; 2 Division of Tuberculosis Elimination, Centers for Disease Control and Prevention, Atlanta, Georgia, United States of America; 3 International Emerging Infections Program, Thailand Ministry of Public Health – U.S. Centers for Disease Control and Prevention Collaboration, Nonthaburi, Thailand; McGill University, Canada

## Abstract

**Background:**

Among approximately 163.5 million foreign-born persons admitted to the United States annually, only 500,000 immigrants and refugees are required to undergo overseas tuberculosis (TB) screening. It is unclear what extent of the unscreened nonimmigrant visitors contributes to the burden of foreign-born TB in the United States.

**Methodology/Principal Findings:**

We defined foreign-born persons within 1 year after arrival in the United States as “newly arrived”, and utilized data from U.S. Department of Homeland Security, U.S. Centers for Disease Control and Prevention, and World Health Organization to estimate the incidence of TB among newly arrived foreign-born persons in the United States. During 2001 through 2008, 11,500 TB incident cases, including 291 multidrug-resistant TB incident cases, were estimated to occur among 20,989,738 person-years for the 1,479,542,654 newly arrived foreign-born persons in the United States. Of the 11,500 estimated TB incident cases, 41.6% (4,783) occurred among immigrants and refugees, 36.6% (4,211) among students/exchange visitors and temporary workers, 13.8% (1,589) among tourists and business travelers, and 7.3% (834) among Canadian and Mexican nonimmigrant visitors without an I-94 form (e.g., arrival-departure record). The top 3 newly arrived foreign-born populations with the largest estimated TB incident cases per 100,000 admissions were immigrants and refugees from high-incidence countries (e.g., 2008 WHO-estimated TB incidence rate of ≥100 cases/100,000 population/year; 235.8 cases/100,000 admissions, 95% confidence interval [CI], 228.3 to 243.3), students/exchange visitors and temporary workers from high-incidence countries (60.9 cases/100,000 admissions, 95% CI, 58.5 to 63.3), and immigrants and refugees from medium-incidence countries (e.g., 2008 WHO-estimated TB incidence rate of 15–99 cases/100,000 population/year; 55.2 cases/100,000 admissions, 95% CI, 51.6 to 58.8).

**Conclusions/Significance:**

Newly arrived nonimmigrant visitors contribute substantially to the burden of foreign-born TB in the United States. To achieve the goals of TB elimination, direct investment in global TB control and strategies to target nonimmigrant visitors should be considered.

## Introduction

Global migration has a substantial impact on the epidemiology of tuberculosis (TB) in the United States [1]. During 2001 through 2008, 54.6% (62,364) of the 114,323 new TB cases in the United States were diagnosed among foreign-born persons [2]. The rate of TB disease among foreign-born persons varies by duration of time in the United States [3], [4]. The highest rate is among foreign-born persons who have resided in the United States for 1 year or less (121.0 cases per 100,000 population), following by those residing in the United States for 1–5 years (30.0 cases per 100,000 population), and those residing in the United States for more than 5 years (11.9 cases per 100,000 population) [4].

Based on their visa status at the time of arrival, foreign-born persons may be classified as ‘immigrants and refugees’ who intend to reside permanently in the United States, and ‘nonimmigrant visitors’ who stay in the United States temporarily (e.g., students, exchange visitors, temporary workers, tourists, and business travelers) [5]. Approximately 500,000 immigrants and refugees, 163 million nonimmigrant visitors, and an unknown number of unauthorized visitors are admitted to the United States annually [5]–[7]. Among these newly arrivals, only immigrants and refugees are required to undergo screening for TB prior to their arrival in the United States [8]. Overseas TB screening in immigrants and refugees is a relatively high-yield intervention [9]–[11]. During the following-up evaluations after arrival in the United States, active TB is diagnosed in 7.0% of immigrants and refugees with an overseas diagnosis of smear negative TB (e.g., a chest radiograph was suggestive of active TB but sputum smears were negative for acid-fast bacilli on 3 consecutive days), and in 1.6% of those with an overseas diagnosis of inactive TB (e.g., a chest radiograph was suggestive of TB that was no longer clinically active) [9]. Many nonimmigrant visitors are from countries with a high incidence of TB and can stay in the United States for a relatively long period of time [5], [8]. Due to unavailable data for immigration status of foreign-born TB cases in the U.S. National TB Surveillance System [2], it is unclear what extent of the unscreened nonimmigrant visitors contributes to the burden of foreign-born TB in the United States. We utilized data from the U.S. Department of Homeland Security (DHS), the U.S. Centers for Disease Control and Prevention (CDC), and the World Health Organization (WHO), to estimate the incidence of TB among foreign-born persons who had resided for 1 year or less after arrival in the United States during 2001 through 2008.

## Methods

### Population of newly arrived foreign-born persons

The length of stay for foreign-born persons admitted to the United States varies, from a few days to multiple years [12], [13]. In this analysis, we focused on the first year after arrival in the United States, and defined foreign-born persons within 1 year after arrival in the United States as “newly arrived”. We restricted this analysis to newly arrived foreign-born persons since 1) rate of TB disease is highest among foreign-born persons within 1 year after arrival in the United States [3], [4], and 2) this is the time period that would most likely be impacted by potential interventions such as overseas TB screening with follow-up evaluations after arrival in the United States. We included immigrants, refugees, and nonimmigrant visitors, but did not include unauthorized visitors due to unavailable data of annual arrivals by country of citizenship [7]. All nonimmigrant visitors, with an exception of Canadian citizens and some Mexican citizens who have a Border Crossing Card (BCC), are required to submit an arrival-departure record or I-94 form that records the dates of arrival and departure at the ports of entry to the United States. We classified newly arrived foreign-born persons into two categories: 1) immigrants and refugees, and 2) nonimmigrant visitors. We further classified nonimmigrant visitors into 4 visa categories: 1) students/exchange visitors and temporary workers, and their family members (U.S. visa types F1 to F3, J1, J2, M1 to M3, E1 to E3, I1, H1B, H1B1, H1C, H2A, H2B, H2R, H3, H4, L1, L2, O1 to O3, P1 to P4, Q1, R1, R2, TD, and TN), 2) tourists and business travelers (U.S. visa types B1 and B2, and visa waiver programs GB, GT, WB, and WT), 3) diplomats and other representatives, and their family members (U.S. visa types A1 to A3, G1 to G5, and N1 to N7), and 4) Canadian and Mexican nonimmigrant visitors without an I-94 form [5], [6], [14]–[17].

Data of annual admissions for immigrants, and nonimmigrant visitors who were required to submit an I-94 form at the ports of entry during 2001 through 2008 was requested and obtained from DHS. Data of annual admissions for Canadian and Mexican nonimmigrant visitors without an I-94 form during the same period were also from DHS [14]–[17]. DHS has reported that 28.5% and 71.5% of the nonimmigrant visitors without an I-94 form are from Canada and Mexico, respectively [12]. We applied these proportions to estimate annual admissions of Canadian and Mexican nonimmigrant visitors without an I-94 form. Data of annual admissions for refugees was obtained from CDC's TB notification system in immigrants and refugees [9]. We excluded 911,490 admissions of foreign-born persons with an unknown country of citizenship, and 12,638 admissions of foreign-born persons with an unknown TB incidence rate for their country of citizenship.

### Country-specific incidence rates of TB and proportions of MDR-TB in new TB cases

Annual estimates of country-specific TB incidence rates (all forms, per 100,000 population per year) from 2001 through 2008, and the estimates of country-specific MDR-TB proportions in new TB cases in 2004 was obtained from WHO [18], [19].

### Country of citizenship of newly arrived foreign-born persons

We assumed country of birth to be the same as the country of citizenship since data was available only for the country of citizenship for nonimmigrant visitors and country of birth for immigrants and refugees. We classified the country of citizenship as a “high-incidence countries”, “medium-incidence countries”, and “low-incidence countries” when the WHO-estimated country-specific TB incidence rate (all forms) in 2008 was 100 or more cases, 15–99 cases, and 0–14 cases per 100,000 population per year, respectively [18].

### Statistical Analysis

In this analysis, we were interested in incident cases, not prevalent cases, since the number of incident cases could be estimated by accounting for both the admissions of foreign-born persons and their lengths of stay in the United States.

#### 1) Estimates of person-years for newly arrived foreign-born persons

In a specific year during the study period, foreign-born persons likely had a stay of <1 year in the United States after arrival [12], [13]. To estimate the number of TB incident cases among foreign-born persons within 1 year after their arrival, we applied a person-year methodology to account for both the admissions and their lengths of stay in the United States [12]. The number of person-years was calculated as the number of admissions multiplied by their length of stay in the United States. For example, 1 admission with a stay of 12 months would make 1 person-year of continued presence in the United States. On the other hand, 6 admissions with a stay of 2 months for each admission would also make 1 person-year of continued presence in the United States.

We assumed a stay of at least 1 year after arrival for immigrants and refugees, and therefore their number of person-years was equal to the number of their admissions. We applied the DHS-estimated median length of stay to calculate the number of person-years for nonimmigrant visitors with an I-94 form, since 1) the uniform distribution method was likely infeasible since DHS has only reported the frequency distributions for length of stay of <6 months, 6–11 months, and ≥12 months, and 2) the mean length of stay was strongly influenced by those who stayed for a long period of time [13]. The DHS-estimated median length of stay was 109 days for students/exchange visitors and temporary workers, 7 days for tourists and business travelers, and 19 days for diplomats and other representatives [13]. Stratified by country of citizenship and year of arrival, the number of person-years was estimated as [109 days/(365 days/year)]*the number of admissions for students/exchange visitors and temporary workers, [7 days/(365 days/year)]*the number of admissions for tourists and business travelers, and [19 days/(365 days/year)]*the number of admissions for diplomats and other representatives.

DHS has only reported the mean length of stay for nonimmigrant visitors without an I-94 form (1.1 days for those from Mexico and 3.7 days for those from Canada) [12]. Stratified by year of arrival, the number of person-years for nonimmigrant visitors without an I-94 form was estimated as [1.1 days/(365 days/year)]*the number of admissions for those from Mexico, and [(3.7 days/(365 days/year)]*the number of admissions for those from Canada.

#### 2) Estimates of incident cases of TB and MDR-TB among newly arrived foreign-born persons

We were unable to accurately estimate country-specific TB incidence rate for foreign-born persons within 1 year after their arrival in the United States by using the data from the U.S. National TB Surveillance System since 1) cases reported to the system included prevalent cases, 2) cases among foreign-born persons with a short length of stay were less likely to seek care in the United States, and therefore might not be reported to the system, 3) cases among unauthorized visitors were reported to the system, but annual arrivals of unauthorized visitors were unknown [7], and 4) immigration status was unavailable for cases reported to the system [2]. For foreign-born persons, the exposure to TB mycobacterium likely occurred in their country of origin, and therefore the incidence rate among foreign-born persons within the first year after their arrival in the United States would be most similar to the incidence rate in their country of origin [4]. In this analysis, we assumed that in a specific year, newly arrived foreign-born persons had the WHO-estimated TB incident rate for their country of citizenship in the same year. Stratified by visa category, country of citizenship, and year of arrival, the number of TB incident cases occurring among newly arrived foreign-born persons was estimated as the person-years in a specific year*the WHO-estimated country-specific TB incidence rate in the same year.

WHO has reported country-specific proportions of MDR-TB in new TB cases for 2004, 2007, and 2008 [19]–[21]. Since MDR-TB proportions in new TB cases increased over time, we applied the WHO-estimated country-specific proportions in 2004 (the mid-study period) to avoid over-estimation. Stratified by visa category and country of citizenship, the number of MDR-TB incident cases occurring among newly arrived foreign-born persons was calculated as the estimated number of TB incident cases during 2001–2008*the 2004 WHO-estimated country-specific MDR-TB proportion in new TB cases.

To compare the impact of newly arrived foreign-born populations on foreign-born TB in the United States, we calculated the estimated numbers of TB and MDR-TB incident cases per 100,000 person-years, as well as the estimated numbers of TB and MDR-TB incident cases per 100,000 admissions. To assess the accuracy of our estimates, we compared the estimated numbers of cases to the actual numbers of cases reported to the U.S. National TB Surveillance System during the study period [2].

## Results

Of 1,479,542,654 admissions of foreign-born persons to the United States from 2001 through 2008, 0.2% (3,625,619) were immigrants and refugees, 1.5% (22,328,345) were students/exchange visitors and temporary workers, 15.6% (230,673,431) were tourists and business travelers, 0.1% (2,215,260) were diplomats and other representatives, and 82.5% (1,220,700,000) were Canadian and Mexican nonimmigrant visitors without an I-94 form (Table 1). We estimated that 11,500 TB incident cases, including 291 MDR-TB incident cases, occurred among 20,989,738 person-years for the 1,479,542,654 newly arrived foreign-born persons (Table 1). During the same period, the U.S. National TB Surveillance System has reported that 12,099 TB cases, including 249 MDR-TB cases, were diagnosed among foreign-born persons within 1 year after their arrival in the United States.

**Table 1:** Estimates of incident cases of TB and MDR-TB among newly arrived foreign-born persons in the United States, 2001–2008.*

Visa category	Admissions (%)	Person-years (%)†	TB†			MDR-TB†		
			no. (%)	no./100,000 person-years (95% CI)	no./100,000 admissions (95% CI)	no. (%)	no./100,000 person-years (95% CI)	no./100,000 admissions (95% CI)
Immigrant and refugee‡	3,625,619 (0.2)	3,625,619 (17.3)	4,783 (41.6)	131.9 (128.2, 135.7)	131.9 (128.2, 135.7)	127 (43.6)	3.5 (2.9, 4.1)	3.5 (2.9, 4.1)
Student/exchange visitor and temporary worker	22,328,345 (1.5)	6,667,917 (31.8)	4,211 (36.6)	63.1 (61.2, 65.1)	18.9 (18.3, 19.4)	111 (38.1)	1.7 (1.4, 2.0)	0.5 (0.4, 0.6)
Tourist and business traveler	230,673,431 (15.6)	4,423,874 (21.1)	1,589 (13.8)	35.9 (34.1, 37.7)	0.7 (0.6, 0.7)	34 (11.7)	0.8 (0.5, 1.0)	<0.1
Diplomat and other representative	2,215,260 (0.1)	115,315 (0.5)	84 (0.7)	72.5 (56.8, 88.9)	3.8 (3.0, 4.6)	2 (0.7)	1.7 (0, 4.6)	0.1 (0.0, 0.2)
Canadian and Mexican nonimmigrant visitor without an I-94 form	1,220,700,000 (82.5)	6,157,014 (29.3)	834 (7.3)	13.5 (12.6, 14.5)	0.1 (0.1, 0.1)	17 (5.8)	0.3 (0.1, 0.4)	<0.1
**Total**	1,479,542,654 (100.0)	20,989,738 (100.0)	11,500 (100.0)	54.7 (53.8, 55.8)	0.8 (0.8, 0.8)	291 (100.0)	1.4 (1.2, 1.6)	<0.1
**Per year**	184,942,832	2,623,717	1,438			36		

*Newly arrived foreign-born persons are those who have resided in the United States for up to 1 year after their arrival.

† See the Methods Section for details of the estimations.

‡ The number of person-years is the same as the number of admissions, since immigrants and refugees are assumed to stay in the United States for at least 1 year after their arrival.

Of the 11,500 estimated TB incident cases, 41.6% (4,783) occurred among immigrants and refugees, 36.6% (4,211) among students/exchange visitors and temporary workers, 13.8% (1,589) among tourists and business travelers, 0.7% (84) among diplomats and other representatives, and 7.3% (834) among Canadian and Mexican nonimmigrant visitors without an I-94 form (Table 1). Overall, 1.3% of the 1,479,542,654 admissions of foreign-born persons were from high-incidence countries, but they accounted for 60.9% of the 11,500 estimated TB incident cases among the newly arrived foreign-born persons. Only 44.8% of the 3,625,619 admissions of immigrants and refugees were from high-incidence countries, but they accounted for 80.0% of the 4,783 estimated TB incident cases among the newly arrived immigrants and refugees (Table 2); 18.7% of the 22,328,345 admissions of students/exchange visitors and temporary workers were from high-incidence countries, but they accounted for 60.4% of the 4,211 estimated TB incident cases among the newly arrived students/exchange visitors and temporary workers (Table 3); 5.4% of the 230,673,431 admissions of tourist and business travelers were from high-incidence countries, but they accounted for 36.4% of the 1,589 estimated TB incident cases among the newly arrived tourists and business travelers (Table 4).

**Table 2:** Estimates of incident cases of TB and MDR-TB among newly arrived immigrants and refugees in the United States, 2001–2008.*

Variable	Admissions or person-years (%)†	TB‡		MDR-TB‡	
		no. (%)	no./100,000 person-years or admissions (95% CI)	no. (%)	no./100,000 person-years or admissions (95% CI)
**Incidence of TB in country of citizenship** §					
Low-incidence (0–14 cases/100,000)	319,499 (8.8)	27 (0.6)	8.3 (5.1, 11.8)	<1 (0.2)	0.1 (0.0, 0.2)
Medium-incidence (15–99 cases/100,000)	1,683,084 (46.4)	929 (19.4)	55.2 (51.6, 58.8)	38 (29.7)	2.2 (1.5, 3.0)
High-incidence (≥100 cases/100,000)	1,623,036 (44.8)	3,827 (80.0)	235.8 (228.3, 243.3)	89 (70.1)	5.5 (4.3, 6.7)
**Country of citizenship** ¶					
Philippines	289,332 (8.0)	876 (18.3)	302.7 (282.6, 323.0)	13 (10.3)	4.5 (1.9, 7.1)
India	226,351 (6.2)	380 (8.0)	168.0 (150.8, 185.0)	9 (7.2)	4.0 (1.2, 6.8)
Vietnam	165,935 (4.6)	339 (7.1)	204.1 (182.3, 226.3)	8 (6.1)	4.7 (1.2, 8.5)
China#	309,336 (8.5)	310 (6.5)	100.3 (88.9, 111.5)	16 (12.9)	5.3 (2.5, 7.9)
Haiti	88,699 (2.4)	236 (4.9)	266.3 (231.6, 300.5)	3 (2.6)	3.7 (0.0, 7.8)
Ethiopia	57,973 (1.6)	223 (4.7)	384.7 (333.4, 435.9)	4 (3.0)	6.5 (0.0, 14.5)
Nigeria	49,626 (1.4)	156 (3.3)	315.3 (264.1, 364.6)	3 (2.1)	5.4 (0.0, 13.9)
Pakistan	64,354 (1.8)	149 (3.1)	231.0 (193.6, 269.4)	3 (2.2)	4.4 (0.0, 10.7)
Burma	34,974 (1.0)	141 (3.0)	404.0 (335.3, 471.0)	6 (4.9)	17.8 (2.0, 32.3)
Dominican Republic	164,302 (4.5)	137 (2.9)	83.3 (69.1, 97.6)	9 (7.1)	5.5 (1.6, 9.4)
Other countries	2,174,739 (60.0)	1,835 (38.4)	84.4 (80.5, 88.3)	53 (41.5)	2.4 (1.7, 3.1)
**Total**	3,625,619 (100.0)	4,783 (100.0)	131.9 (128.2, 135.7)	127 (100.0)	3.5 (2.9, 4.1)
**Per year**	453,202	598		16	

*Newly arrived immigrants and refugees are those who have resided in the United States for up to 1 year after their arrival.

† The number of person-years is the same as the number of admissions, since immigrants and refugees are assumed to stay in the United States for at least 1 year after their arrival.

‡ See the Methods Section for details of the estimations.

§ Values are World Health Organization estimates for 2008.

¶ Countries are listed in descending order, according to the estimated number of TB incident cases. Country of citizenship is assumed to be the same as country of birth for immigrants and refugees.

# The values for China include those for Hong Kong, Macau, and Taiwan.

**Table 3:** Estimates of incident cases of TB and MDR-TB among newly arrived students/exchange visitors and temporary workers in the United States, 2001–2008.*

Variable	Admissions (%)	Person-years (%)†	TB†			MDR-TB†		
			no. (%)	no./100,000 person-years (95% CI)	no./100,000 admissions (95% CI)	no. (%)	no./100,000 person-years (95% CI)	no./100,000 admissions (95% CI)
**Incidence of TB in country of citizenship** ‡								
Low-incidence (0–14 cases/100,000)	7,973,041(35.7)	2,380,990 (35.7)	212 (5.0)	8.9 (7.7, 10.1)	2.7 (2.3, 3.0)	2 (1.7)	0.1 (0.0, 0.2)	<0.1
Medium-incidence (15–99 cases/100,000)	10,178,764 (45.6)	3,039,686 (45.6)	1,454 (34.5)	47.8 (45.4, 50.3)	14.3 (13.6, 15.0)	42 (37.7)	1.4 (1.0, 1.8)	0.4 (0.3, 0.5)
High-incidence (≥100 cases/100,000)	4,176,540 (18.7)	1,247,241 (18.7)	2,544 (60.4)	204.0 (196.0, 211.9)	60.9 (58.5, 63.3)	67 (60.6)	5.4 (4.1, 6.7)	1.6 (1.2, 2.0)
**Country of citizenship** §								
India	2,290,171 (10.3)	683,914 (10.3)	1,149 (27.3)	168.0 (158.2, 177.8)	50.2 (47.3, 53.1)	28 (24.9)	4.0 (2.5, 5.7)	1.2 (0.8, 1.7)
China¶	1,249,637 (5.6)	373,179 (5.6)	373 (8.9)	100.0 (89.7, 110.2)	29.8 (26.8, 32.9)	20 (17.9)	5.3 (2.9, 7.8)	1.6 (0.9, 2.3)
South Africa	140,149 (0.6)	41,853 (0.6)	362 (8.6)	864.7 (775.0, 954.8)	258.2 (231.4, 285.2)	7 (5.9)	15.6 (3.1, 30.3)	4.6 (0.9, 9.1)
South Korea	1,294,260 (5.8)	386,505 (5.8)	342 (8.1)	88.4 (79.0, 98.0)	26.4 (23.6, 29.3)	8 (6.8)	1.9 (0.5, 3.6)	0.6 (0.2, 1.1)
Philippines	176,815 (0.8)	52,802 (0.8)	159 (3.8)	301.6 (253.4, 348.8)	90.1 (75.7, 104.2)	2 (2.2)	4.5 (0.0, 10.0)	1.4 (0.0, 3.0)
Japan	1,990,023 (8.9)	594,281 (8.9)	156 (3.7)	26.3 (22.1, 30.5)	7.8 (6.6, 9.1)	1 (1.3)	0.2 (0.0, 0.6)	0.1 (0.0, 0.2)
Mexico	2,016,211 (9.0)	602,101 (9.0)	136 (3.2)	22.5 (18.7, 26.5)	6.7 (5.6, 7.9)	3 (2.9)	0.5 (0.0, 1.2)	0.2 (0.0, 0.3)
Russia	313,406 (1.4)	93,592 (1.4)	101 (2.4)	108.2 (86.4, 129.5)	32.3 (25.8, 38.7)	10 (9.1)	10.8 (3.5, 17.8)	3.2 (1.1, 5.3)
Brazil	516,154 (2.3)	154,139 (2.3)	80 (1.9)	51.8 (40.2, 63.6)	15.5 (12.0, 19.0)	1 (0.6)	0.5 (0.0, 2.2)	0.1 (0.0, 0.7)
Thailand	172,413 (0.8)	51,488 (0.8)	71 (1.7)	137.0 (104.09, 170.9)	40.9 (31.3, 51.1)	1 (0.6)	1.2 (0.0, 11.4)	0.4 (0.0, 3.4)
Other countries	12,169,108 (54.5)	3,634,062 (54.5)	1,282 (30.5)	35.3 (33.3, 37.2)	10.5 (10.0, 11.1)	31 (27.8)	0.8 (0.5, 1.2)	0.3 (0.2, 0.4)
**Total**	22,328,345 (100.0)	6,667,917 (100.0)	4,211 (100.0)	63.1 (61.2, 65.1)	18.9 (18.3, 19.4)	111 (100.0)	1.7 (1.4, 2.0)	0.5 (0.4, 0.6)
**Per year**	2,791,043	833,490	526			14		

*Newly arrived students/exchange visitors and temporary workers are those who have resided in the United States for up to 1 year after their arrival.

† See the Methods Section for details of the estimations.

‡ Values are World Health Organization estimates for 2008.

§ Countries are listed in descending order, according to the number of estimated incident cases of TB.

¶ The values for China include those for Hong Kong, Macau, and Taiwan.

**Table 4:** Estimates of incident cases of TB and MDR-TB among newly arrived tourists and business travelers in the United States, 2001–2008.*

Variable	Admissions (%)	Person-years (%)†	TB†			MDR-TB†		
			no. (%)	no./100,000 person-years (95% CI)	no./100,000 admissions (95% CI)	no. (%)	no./100,000 person-years (95% CI)	no./100,000 admissions (95% CI)
**Incidence of TB in country of citizenship** ‡								
Low-incidence (0–14 cases/100,000)	101,126,330 (43.8)	1,939,409 (43.8)	199 (12.5)	10.3 (8.8, 11.7)	0.2 (0.2,0.2)	2 (5.2)	0.1 (0.0, 0.3)	<0.1
Medium-incidence (15–99 cases/100,000)	117,140,427 (50.8)	2,246,529 (50.8)	812 (51.1)	36.1 (33.6, 38.7)	0.7 (0.6, 0.7)	20 (57.3)	0.9 (0.5, 1.3)	<0.1
High-incidence (≥100 cases/100,000)	12,406,675 (5.4)	237,936 (5.4)	578 (36.4)	242.9 (222.9, 262.9)	4.7 (4.3, 5.0)	13 (37.5)	5.4 (2.3, 8.6)	0.1 (0.0, 0.2)
**Country of citizenship** §								
Mexico	40,969,988 (17.8)	785,726 (17.8)	181 (11.4)	23.1 (19.6, 26.5)	0.4 (0.4, 0.5)	4 (12.7)	0.6 (0.0, 1.1)	<0.1
Japan	30,789,951 (13.3)	590,492 (13.3)	155 (9.8)	26.3 (22.0, 30.5)	0.5 (0.4, 0.6)	1 (4.1)	0.2 (0.0, 0.6)	<0.1
South Africa	736,984 (0.3)	14,134 (0.3)	124 (7.8)	877.4 (720.0, 1,034.6)	16.8 (13.8, 19.9)	2 (6.5)	15.8 (0.0, 37.3)	0.3 (0.0, 0.7)
China¶	5,583,994 (2.4)	107,090 (2.4)	107 (6.7)	100.1 (80.5, 119.3)	1.9 (1.5, 2.3)	6 (16.6)	5.3 (0.7, 10.6)	0.1 (0.0, 0.2)
India	3,294,863 (1.4)	63,189 (1.4)	106 (6.7)	168.0 (135.1, 200.5)	3.2 (2.6, 3.8)	3 (7.4)	4.0 (0.0, 10.9)	0.1 (0.0, 0.2)
United Kingdom	37,571,301 (16.3)	720,545 (16.3)	98 (6.2)	13.7 (10.8, 16.4)	0.3 (0.2, 0.3)	1 (2.6)	0.1 (0.0, 0.5)	<0.1
South Korea	5,702,310 (2.5)	109,359 (2.5)	97 (6.1)	88.4 (70.6, 106.8)	1.7 (1.4, 2.1)	2 (6.2)	1.9 (0.0, 4.8)	<0.1
Philippines	1,537,487 (0.7)	29,486 (0.7)	90 (5.6)	303.5 (240.6, 369.9)	5.8 (4.6, 7.1)	1 (3.9)	4.6 (0.0, 11.7)	0.1 (0.0, 0.2)
Brazil	4,331,167 (1.9)	83,063 (1.9)	43 (2.7)	51.5 (35.7, 67.8)	1.0 (0.7, 1.3)	<1 (1.1)	0.5 (0.0, 0.6)	<0.1
Peru	1,424,133 (0.6)	27,312 (0.6)	40 (2.5)	145.6 (99.3, 193.6)	2.8 (1.9, 3.7)	1 (3.5)	4.4 (0.0, 12.7)	0.1 (0.0, 0.2)
Other countries	98,731,255 (42.8)	1,893,476 (42.8)	548 (34.5)	28.9 (26.5, 31.4)	0.6 (0.5, 0.6)	12 (35.5)	0.6 (0.3, 1.0)	<0.1
**Total**	230,673,431 (100.0)	4,423,874 (100.0)	1,589 (100.0)	35.9 (34.1, 37.7)	0.7 (0.6, 0.7)	34 (100.0)	0.8 (0.5, 1.0)	<0.1
**Per year**	28,834,179	552,984	199			4		

*Newly arrived tourist and business travelers are those who have resided in the United States for up to 1 year after their arrival.

† See the Methods section for details of the estimations.

‡ Values are World Health Organization estimates for 2008.

§ Countries are listed in descending order, according to the number of estimated incident cases of TB.

¶ The values for China include those for Hong Kong, Macau, and Taiwan.

Only 29.8% of immigrants and refugees were from the top 5 countries (the Philippines, India, Vietnam, China, and Haiti), but they accounted for 44.8% of the estimated TB incident cases among the newly arrived immigrants and refugees (Table 2); 23.1% of students/exchanges visitors and temporary workers were from the top 5 countries (India, China, South Africa, South Korea, and the Philippines), but they accounted for 56.6% of the estimated TB incident cases among the newly arrived students/exchange visitors and temporary workers (Table 3); 35.3% of tourists and business travelers were from the top 5 countries (Mexico, Japan, South Africa, China, and India), but they accounted for 42.4% of the estimated TB incident cases among the newly arrived tourists and business travelers (Tables 4).

**Figure 1:**
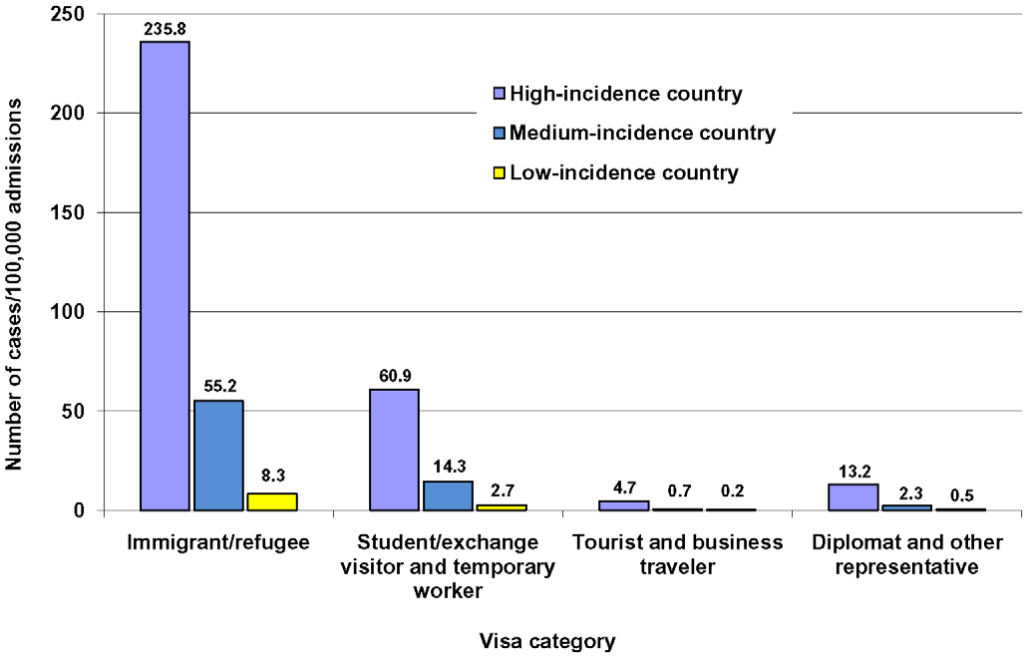
Estimated number of TB incident cases per 100,000 admissions among newly arrived foreign-born populations in the United States, 2001–2008.* * 1) Newly arrived foreign-born persons are those who have resided in the United States for up to 1 year after arrival; 2) low, medium, and high-incidence country are countries of citizenship where the 2008 WHO-estimated TB incidence rate is 0–14 cases, 15–99 cases, and ≥100 cases per 100,000 population per year, respectively; 3) Canadian and Mexican nonimmigrant visitors without an I-94 form (not shown): 0.07 cases/100,000 admissions for those from Mexico, and 0.06 cases/100,000 admissions for those from Canada.

The top 5 newly arrived foreign-born populations with the largest number of estimated TB incident cases per 100,000 admissions were immigrants and refugees from high-incidence countries (235.8 cases per 100,000 admissions, 95% confidence interval [CI], 228.3 to 243.3), students/exchange visitors and temporary workers from high-incidence countries, 60.9 cases per 100,000 admissions (95% CI, 58.5 to 63.3), immigrants and refugees from medium-incidence countries (55.2 cases per 100,000 admissions, 95% CI, 51.6 to 58.8), students/exchange visitors and temporary workers from medium-incidence countries (14.3 cases per 100,000 admissions, 95% CI, 13.6 to 15.0), and diplomats and other representatives from high-incidence countries (13.2 cases per 100,000 admissions, 95% CI, 9.8 to 16.7; Tables 2, 3, 4 & Figure 1).

## Discussion

We estimated that newly arrived nonimmigrant visitors contribute substantially to the burden of foreign-born TB in the United States. From 2001 through 2008, on average, 1,438 TB incident cases per year were estimated to occur among newly arrived foreign-born persons in the United States. Of the 1,438 annual estimated TB incident cases, 41.6% (598) were among immigrants and refugees who were screened for TB overseas, and 58.4% (840) were among nonimmigrant visitors who were not screened for TB overseas. To reduce the incidence of TB in newly arrived foreign-born populations in the United States, strategies to target nonimmigrant visitors should be considered. Such strategies may include screening for TB in nonimmigrant visitors if they are from high-incidence countries and also intend to stay in the United States for a long period of time.

Overseas TB screening is a relatively high-yield intervention [9]–[11], but it is only required for U.S.-bound immigrants and refugees [8]. This intervention consists of two integrated components, screening for TB overseas and follow-up evaluations after arrival in the United States [9], [10]. One objective of overseas TB screening is to prevent the importation of TB into the United States, and the other objective is to identify immigrants and refugees with suspected TB for follow-up evaluations in the United States [9]. A recent study has reported that during follow-up evaluations in the United States, active pulmonary TB is diagnosed in 7.0% of immigrants and refugees with an overseas diagnosis of smear-negative TB and 1.6% of those with an overseas diagnosis of inactive TB [9]. Due to the large volume of annual admissions and the overall low TB rate, screening for TB in nonimmigrant visitors to the United States is considered to be infeasible and of low-yield [22], [23]. However, this intervention could be effective if it targets those who are from countries with a high incidence of TB, particularly those who also intend to stay in the United States for a long time period. Since overseas TB screening in approximately 400,000 immigrants and refugees per year has been feasible and effective [8]–[10], we considered the potential impact of screening for TB in selected nonimmigrant visitors. For example, during 2001 through 2008, there were approximately 522,000 annual admissions of students/exchange visitors and temporary workers per year were from high-incidence countries, and they accounted for 22.1% of the 1,438 annual estimated TB incident cases in the newly arrived foreign-born persons. In comparison, there were approximately 203,000 annual admissions of immigrants and refugees from high-incidence countries and 210,000 annual admissions from medium-incidence countries, and they accounted for 33.2% and 8.1% of the 1,438 annual estimated TB incident cases in the newly arrived foreign-born persons, respectively. Furthermore, newly arrived students/exchange visitors and temporary workers from high-incidence countries had a larger number of estimated TB incident cases per 100,000 admissions than any other newly arrived foreign-born populations except immigrants and refugees from high-incidence countries. Post-arrival follow-up evaluations of students/exchange visitors and temporary workers with suspected TB is likely possible since they stay in the United States for an average of 180 days [13].

From 2001 through 2008, on average, 28.8 million of tourists and business travelers per year were admitted to the United States, but they accounted for 13.8% of the 1,438 annual estimated TB incident cases in the newly arrived foreign-born persons. Due to the large annual admissions and a small number of estimated TB incident cases per 100,000 admissions, intervention of TB in tourists and business travelers is likely to be infeasible and of low-yield. Newly arrived diplomats and other representatives likely had a small impact on foreign-born TB in the United States. Despite the large volume of admissions, Canadian and Mexican nonimmigrant visitors without an I-94 form only accounted for 7.3% of the estimated TB incident cases among the newly arrived foreign-born persons.

There are several barriers to implement the potential intervention for nonimmigrant visitors to the United States. Firstly, it increases the cost and workload for federal governmental agencies, as well as state and local health departments. Secondly, requiring proof of TB screening of nonimmigrant visitors would significantly deviate from the U.S. open-door policy and would have great logistical and political implications [22]. Finally, many high-incidence countries may not have the facility and capacity to implement the TB screening program for U.S.-bound nonimmigrant visitors.

Canada and some European countries require TB screening for long-term nonimmigrant visitors from medium and high-incidence countries [24], [25]. In the United States, some nonimmigrant visitors have TB screening after arrival [26]–[29]. Approximately 600,000 nonimmigrant visitors who have been living in the United States for at least 1 year apply to adjust their status to U.S. permanent residence annually and are required to undergo TB screening [5], [8], [26]–[28]. Some U.S. universities and colleges require international students to have TB screening [29]. Despite these efforts, the TB rate among newly arrived foreign-born persons remains very high in the United States [3], [4], indicating additional TB interventions are needed.

We did not predict the potential yield of screening latent TB infection (LTBI). The current algorithm for overseas TB screening in U.S.-bound immigrants and refugees focuses on preventing the importation of active TB [30], [31]. However, this analysis estimated that there were 1,438 TB incident cases occurred among newly arrived foreign-born persons annually, indicating that screening and treating LTBI is important. A current study has also recommended that U.S. TB control programs should focus on finding and treating LTBI prior to or shortly arrival in the United States since 83.7% of the U.S. foreign-born TB cases are attributed to reactivation TB [32].

From 2001 through 2008, we estimated that 11,500 TB incident cases, including 291 MDR-TB incident cases, occurred among foreign-born persons within 1 year after arrival in the United States. During the same period, according to the U.S. National TB Surveillance System, 12,099 TB cases, including 249 MDR-TB cases, were diagnosed among foreign-born persons within 1 year after arrival in the United States [2]. However, we should be cautioned for comparing our estimate with the reported cases in U.S. National TB Surveillance System since 1) immigration status of foreign-born TB cases was unavailable in the U.S. National TB Surveillance System, 2) our estimate included cases among foreign-born persons with a short length of stay, but these cases might not seek care in the United States and were less likely to be reported to the system, 3) our estimate included incident cases only, but both incident and prevalent cases were reported to the system, and 4) our estimate did not include incident cases among newly arrived unauthorized visitors, but cases among unauthorized visitors were reported to the system.

We compared our estimates with the results of some previous studies. One study reported that, of 114 foreign-born TB cases diagnosed in Tarrant County, Texas, 59.0% was among immigrants, 28.0% among unauthorized visitors, and 19.0% among nonimmigrant visitors. However, this study included foreign-born persons who had been in the United States for >1 year and also likely included more unauthorized visitors because of the study location [33]. Another study found that, in California, 38.1% of 2,549 foreign-born TB cases within 1 year after arrival in the United States were among immigrants and refugees with an overseas TB diagnosis [34]. A recent study reported that, 4,285 immigrants and refugees with an overseas diagnosis of smear-negative TB and 4,480 immigrants and refugees with an overseas diagnosis of inactive TB were admitted to the United States annually during 1999 through 2005 [9]. Based on the findings in the same study, 372 cases of active pulmonary TB (4,285*7.0%+4,480*1.6%) were likely diagnosed among immigrants and refugees with an overseas TB diagnosis annually. In comparison, we found that immigrants and refugees accounted for 41.6% (598) of the 1,438 annual estimated TB incident cases in newly arrived foreign-born persons. Our estimates were larger since we included TB incident cases in newly arrived immigrants and refugees without an overseas TB diagnosis.

This analysis had several limitations. Firstly, the DHS-estimated length of stay might be biased since DHS used the 2003 departure data for nonimmigrant visitors with an I-94 form and the 2004 departure data for Canadian and Mexican nonimmigrant visitors without an I-94 form in its estimations. Secondly, we did not estimate the incidence of TB among newly arrived unauthorized visitors due to the lack of available data. DHS has reported that, of the estimated 10.8 million unauthorized visitors in January 2009, 4.4% (470,000) were from two high-incidence countries (the Philippines and India) and 79.6% (8.6 million) were from eight medium-incidence countries (Mexico, El Salvador, Guatemala, Honduras, South Korea, Ecuador, Brazil, and China) [7]. DHS has also estimated that 37.0% (4.0 million) of the 10.8 million unauthorized visitors in January 2009 had entered the United States in January 2000 or later [7], but it did not estimate the annual arrivals of unauthorized visitors by country of citizenship. Finally, U.S.-bound immigrants and nonimmigrant visitors likely have better socioeconomic status, therefore might have a lower TB incidence rate than the general population of their country of citizenship. However, a recent study has found that, for foreign-born persons from 9 of the top 10 countries of origin with the largest number of TB cases in the United States, the TB rates among those who have been in the United States for 1 year or less after arrival are actually higher than the TB rates of their countries of origin [4].

Newly arrived immigrants and refugees, as well as nonimmigrant visitors, contribute substantially to the burden of foreign-born TB in the United States. CDC has already implemented modernized TB screening which includes mycobacterial cultures, drug-susceptibility testing, and directly observed therapy for immigrant visa applicants in the Philippines, China, Vietnam, Indian, Mexico, and other countries [31], [35]. To further reduce the incidence of TB in foreign-born populations in the United States, strategies to target nonimmigrant visitors, especially those who are from high-incidence countries and also intend to stay for a long period of time in the United States, should be considered. Cost-effectiveness analyses and studies to better estimate the TB rates in U.S.-bound foreign-born populations could guide decisions about which populations should be targeted.
